# Methylmercury inhibits prolactin release in a cell line of pituitary origin

**DOI:** 10.1590/1414-431X20154165

**Published:** 2015-06-23

**Authors:** L.A.L. Maués, B.M. Macchi, M.E. Crespo-López, L.E. Nasciutti, D.L.W. Picanço-Diniz, J. Antunes-Rodrigues, J.L.M. do Nascimento

**Affiliations:** 1 Laboratório de Neuroquímica Molecular e Celular, Instituto de Ciências Biológicas, Universidade Federal do Pará, Belém, PA, Brasil; 2 Laboratório de Farmacologia Molecular, Instituto de Ciências Biológicas, Universidade Federal do Pará, Belém, PA, Brasil; 3 Laboratório de Neuroendocrinologia, Instituto de Ciências Biológicas, Universidade Federal do Pará, Belém, PA, Brasil; 4 Laboratório de Interações Celulares, Instituto de Ciências Biomédicas, Universidade Federal do Rio de Janeiro, Rio de Janeiro, RJ, Brasil; 5 Departamento de Fisiologia, Faculdade de Medicina de Ribeirão Preto, Universidade de São Paulo, Ribeirão Preto, SP, Brasil

**Keywords:** Mercury, Methylmercury, Prolactin, Oxidative stress, Pituitary, Nitric oxide

## Abstract

Heavy metals, such as methylmercury, are key environmental pollutants that easily reach human beings by bioaccumulation through the food chain. Several reports have demonstrated that endocrine organs, and especially the pituitary gland, are potential targets for mercury accumulation; however, the effects on the regulation of hormonal release are unclear. It has been suggested that serum prolactin could represent a biomarker of heavy metal exposure. The aim of this study was to evaluate the effect of methylmercury on prolactin release and the role of the nitrergic system using prolactin secretory cells (the mammosomatotroph cell line, GH3B6). Exposure to methylmercury (0-100 μM) was cytotoxic in a time- and concentration-dependent manner, with an LC_50_ higher than described for cells of neuronal origin, suggesting GH3B6 cells have a relative resistance. Methylmercury (at exposures as low as 1 μM for 2 h) also decreased prolactin release. Interestingly, inhibition of nitric oxide synthase by N-nitro-L-arginine completely prevented the decrease in prolactin release without acute neurotoxic effects of methylmercury. These data indicate that the decrease in prolactin production occurs via activation of the nitrergic system and is an early effect of methylmercury in cells of pituitary origin.

## Introduction

Heavy metals, such as methylmercury (MeHg), are environmental pollutants that readily affect human beings by bioaccumulation through the food chain (1). Several reports support the idea that the central nervous system represents a major target of mercury (1,2), and endocrine organs may also accumulate high mercury concentrations (3). Studies performed in humans and animal models have demonstrated that individuals exposed to different forms of mercury show a significant mercury concentration in the pituitary gland (4).

The pituitary gland is a critical neuroendocrine organ with a posterior attachment to the hypothalamus. The pituitary anterior lobe (or adenohypophysis) is anatomically different from the hypothalamus and contains a collection of endocrine cells (5). Adenohypophyseal secretory cells include somatotrophs (nearly 50%), which produce somatotropin (growth hormone, GH); corticotrophs (15-20%), which release adrenocorticotropic hormone; gonadotrophs (10-15%), which synthesize luteinizing hormone and follicle stimulating hormone; thyrotrophs (3-5%), which release thyroid stimulating hormone; and lactotrophs (10-25%), which release prolactin (PRL) (5). Disturbances in pituitary physiology result in hypo- or hyper-secretion of these hormones. Although the pituitary gland has already been highlighted as a potential target of mercury accumulation (4), the effects of this metal on the regulation of hormonal release are unclear. Previous studies showed associations (both positive and negative) between serum PRL and mercury exposure (6). This dual effect may be explained by different interactions between mercury species (inorganic and organic) and PRL secretion by the pituitary gland, which is controlled by neurotransmitters such as dopamine. Thus, serum PRL was suggested as a possible biomarker of heavy metal exposure (7); however, the cellular mechanism remains unknown.

PRL is a single chain protein with 199 amino acids and three disulfide bridges (sharing strong structural homology with GH) (8). The major role of PRL is stimulating breast development and milk production. However, more than 300 additional roles have been attributed to PRL, including salt and water homeostasis, cellular growth, and proliferation (8). PRL influences the hypothalamo-pituitary-gonadal axis, inhibiting the secretion of pulsatile gonadotropin release hormone from the hypothalamus and modifying the activity of some steroidogenic enzymes (8). An excess or depletion of PRL secretion is associated with infertility and menstrual irregularity or even complete amenorrhea (9). In men, it causes increased testosterone and sperm production. Moreover, an excess of PRL can provoke galactorrhea (inappropriate milk production) in women and gynecomastia (breast development) in men (9).

PRL release is regulated by different factors including dopamine, thyrotropin releasing hormone, and nitric oxide (NO) (10). In the adenohypophysis, gonadotrophs and folliculostellate cells express neuronal nitric oxide synthase (nNOS) (11). Although NOS is not present in lactotrophs, these cells contain soluble guanylate cyclase, which leads to an increase in the rate of cGMP synthesis and a decrease in PRL release when stimulated by NO (10). However, *in vitro* cultures containing isolated lactotrophs could express NOS (a type of prolactinoma is nNOS positive), suggesting that autocrine modulation may occur in these conditions (11).

Cell lines releasing PRL have been widely used to study the molecular mechanisms underlying the modulation of hormone secretion, including hypothalamic factors, steroids, and thyrotropin releasing hormone (12). The aim of this study was to evaluate the effect of methylmercury on prolactin release and the role of the nitrergic system, using an experimental model of the rat mammosomatotroph cell line, GH3B6 prolactin secretory cells.

## Material and Methods

### Chemicals

Fetal bovine serum and horse serum were obtained from Gibco (UK). HAM-F12 medium, phosphate buffered saline (PBS), streptomycin, penicillin, gentamicin, methylmercury chloride (MeHgCl, 99.8%), N-nitro-L-arginine (L-NARG), 3-[4,5-dimethylthiazol-2-yl]-2,5-diphenyl-tetrazolium bromide (MTT), and all reagents for radioimmunoassays were purchased from Sigma-Aldrich (USA).

### Cell culture

The rat GH3B6 pituitary adenoma cell line was obtained from the American Type Culture Collection (ATCC; USA) and grown at 37°C under 5% CO_2_ in HAM-F12 medium supplemented with 15% horse serum, 2.5% fetal bovine serum, 40 U/mL penicillin, 40 µg/mL streptomycin, and 1 µg/mL gentamicin. Approximately 2.5×10^5^ cells were plated in 22 mm plastic Petri dishes and kept at 37°C under 5% CO_2_ for 72 h before MeHg exposure.

### Methylmercury and N-nitro-L-arginine exposure

Methylmercury chloride was diluted directly with serum-free culture medium. The GH3B6 cell line was incubated with 0-100 µM of MeHg for 2 or 6 h at 37°C under 5% CO_2_. Where required, co-treatment with 3 mM L-NARG, a classic NOS inhibitor, was carried out for the same incubation times. This concentration was previously used for a similar purpose in cultured cells (13). Finally, cells and conditioned medium were collected for cellular viability determination and prolactin assays, respectively.

### Cellular viability determination

Cellular viability was evaluated by MTT assay as previously described (14). In this assay, the active mitochondria of viable cells reduce the colorless tetrazolium salt MTT, forming dark blue insoluble formazan crystals. Control and MeHg-treated cells were washed twice with PBS and incubated for 3 h with 50 μL of MTT stock solution (5 mg/mL) in 500 μL of PBS. After incubation, 50 μL of 2-propanol was added. Formation of formazan was detected at 570 nm and cellular viability was expressed as the percentage of reduced MTT compared with control values.

### Assay of prolactin release

The prolactin concentration was determined in conditioned medium by double-antibody radioimmunoassay. The rat Prolactin RIA Kit was obtained from the National Hormone and Pituitary Program, National Institute of Diabetes and Digestive and Kidney Diseases, USA.

### Statistical analysis

Statistical tests were performed with the INSTAT software (GraphPad, USA). A one-way analysis of variance (ANOVA), followed by Tukey's *post hoc* test when appropriate, was used to compare average values between groups. P<0.05 was considered to be statistically significant.

## Results

### Effect of methylmercury on cellular viability

Exposure to methylmercury produced a significant decrease in cellular viability in a time-dependent manner at concentrations above 10 µM (Supplementary Figure S1). When 100 μM MeHg was used, incubation for 6 h proved to be significantly more toxic than incubation for 2 h (viable cells reduced by approximately 50% and 30%, respectively, compared with the control group; P<0.001). The concentration-response curves were fitted to sigmoid curves designed to calculate LC_50_ values, which were 166.42 μM (R^2^ = 0.983) and 92.64 μM (R^2^ = 0.968) for 2 h and 6 h of incubation, respectively. Based on these data, 1, 10, and 100 μM MeHg were selected for 2 h and 1 and 10 μM for 6 h incubation to result in >70% cell viability.

### Effect of methylmercury on prolactin release

**Figure 1:**
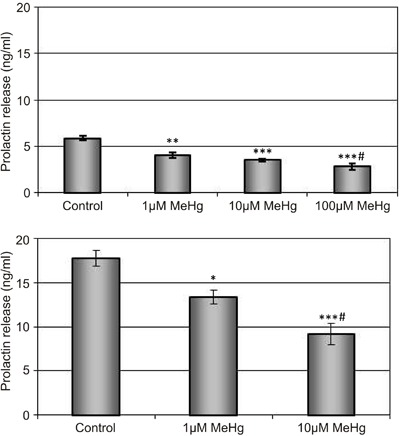
Prolactin release by the rat pituitary cell line GH3B6 exposed to different methylmercury (MeHg) concentrations for 2 h (*top panel*) or 6 h (*bottom panel*). Data are reported as means ± SE (n=6). *P<0.05, **P<0.01, and ***P<0.001 *vs* control; ^#^P<0.05 *vs* the 1-μM group (ANOVA with Tukey's test).

All MeHg concentrations significantly decreased prolactin release from GH3B6 cells (Figure 1). Incubation for 2 h resulted in lower levels of prolactin release than 6 h of incubation. MeHg inhibition of prolactin release was evident even at the lowest concentration (1 μM; P<0.001). After 6 h of MeHg exposure, a significant difference (P<0.05) was detected between the 1- and 10-μM MeHg-treated groups (Figure 1, bottom panel).

### Effect of L-NARG on the inhibition of prolactin release by methylmercury

**Figure 2:**
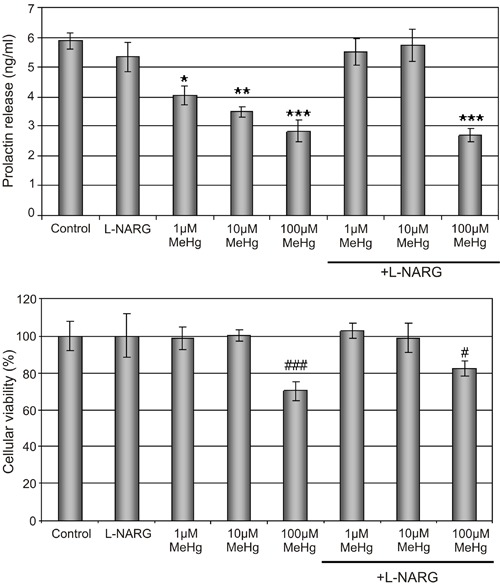
Prolactin release (*top panel*) and cellular viability (*bottom panel*) of the rat pituitary cell line GH3B6 exposed to different methylmercury (MeHg) concentrations and/or 3 mM N-nitro-L-arginine (L-NARG) for 2 h. Data are reported as means ± SE (n=6). *P<0.05, **P<0.01, and ***P<0.001 *vs* control and groups incubated with L-NARG and L-NARG + MeHg (1 and 10 μM); ^#^P<0.05 and ^###^P<0.001 *vs* all groups except those incubated with 100 μM MeHg and L-NARG + 100 μM MeHg (ANOVA with Tukey's test).

**Figure 3:**
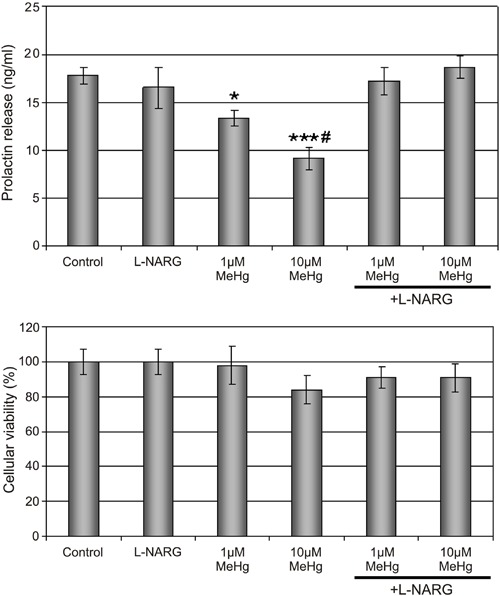
Prolactin release (*top panel*) and cellular viability (*bottom panel*) of the rat pituitary cell line GH3B6 exposed to different methylmercury (MeHg) concentrations and/or 3 mM N-nitro-L-arginine (L-NARG) for 6 h. Data are reported as means ± SE (n=6). *P<0.05 and ***P<0.001 *vs* control and groups incubated with L-NARG and MeHg + L-NARG; ^#^P<0.05 *vs* 1-μM group (ANOVA with Tukey's test).

There were no differences in cellular viability and prolactin release, compared with the control groups, when GH3B6 cells were incubated with 3 mM L-NARG (Figures 2 and 3). Co-incubation of MeHg and L-NARG completely prevented the decrease of prolactin release seen with 1 and 10 μM MeHg (Figures 2 and 3, top panels). However, L-NARG did not show any protective effect against the decreased release of prolactin when cells were exposed to 100 μM MeHg for 2 h (perhaps because of the significant reduction in cellular viability in those treatment groups). There was no significant difference in cellular viability between the other groups (Figures 2 and 3, bottom panels).

## Discussion

This work demonstrates, for the first time, using an *in vitro* approach, that MeHg exposure can significantly decrease prolactin release in cells of pituitary origin. The use of a cell line of neoplastic origin is the usual first step in toxicological studies. Specifically, *in vitro* models have traditionally been used for the analysis of mercury toxicity, especially to highlight cellular mechanisms in the brain (1,2). In this study, MeHg exposure was limited to 2 or 6 h to study relatively rapid effects on prolactin release and to avoid excessive cell death.

MeHg exposure of cells of a mammosomatotroph origin showed a relevant cytotoxic effect only when the highest concentration was used (100 μM). The LC_50_ values found in this study for MeHg toxicity in GH3B6 cells were higher than described elsewhere for astrocytes, neurons, and other cell lines with a central nervous system origin (2). This difference is probably due to longer MeHg incubations in the previous studies (24 h or more). In addition, the LC_50_ values in this study were higher than those reported in a previous study performed in cerebellar granule and retinal cell cultures with the same times of exposure, indicating cells of pituitary origin may have a higher resistance to MeHg.

Interestingly, *in vivo* studies (3,15) demonstrated that the pituitary gland (and especially the anterior pituitary) is one of the organs in which mercury accumulates. For example, high concentrations of mercury in the pituitary gland have been reported in monkeys following long-term subclinical MeHg exposure and in humans exposed to mercury vapor. Despite this distribution, the pituitary gland is not very sensitive to the effects of mercury toxicity when compared with the cerebellum or cortex. Thus, the higher resistance of cells of pituitary origin found in this work could be due to a protective role exerted by PRL against mercury toxicity, especially if one considers that the thiol groups of PRL can be used as scavengers of this metal. (15).

Despite this possible resistance to cellular death, prolactin release in GH3B6 cells was dramatically affected by the two MeHg concentrations used (Figure 1) and decreased in a concentration-dependent manner. Exposure of the cells to 1 μM MeHg for 2 h was sufficient to generate a significant inhibition of prolactin production (reduction of ∼33% when compared with control cells). It is unlikely that extensive apoptosis produced the prolactin decrease observed in this work because Toimela et al. (16) demonstrated no caspase-3 activity in cell lines of CNS origin incubated for 6 h with 10 µM of MeHg. Low MeHg concentrations are able to produce a detectable level of apoptosis, but only after periods of incubation of up to 12 h (16). There may be several phenomena related to the suppression of prolactin release in GH3B6 cells induced by MeHg; however, in the present work, we showed for the first time that the nitrergic system represents an important mediator of prolactin release.

Concentrations of 2.5-10 μM MeHg in the brain (estimated from human blood and hair mercury levels) have been associated with delayed psychomotor development in children and adults with minimal signs of MeHg poisoning (1,17,18). Our results suggest that exposure to similar levels may lead to a decrease in prolactin release from the pituitary gland with consequences for the brain, pointing to the necessity of reviewing the tolerance values (30 µg/L = 30 ppb or 0.03 ppm) published in 1990 by the World Health Organization. Our data contribute to the growing discussions about the safety limits of mercury exposure based on findings that long-term intake of relatively low levels of mercury induced sub-clinical neurobehavioral abnormalities (1,2,18). These early effects may be due to the special sensitivity of the brain and other organs to MeHg toxicity, as described before (1,2) and supported by our data.

The role of prolactin as a potential bioindicator of neurotoxicity in human populations at risk is currently being discussed (7,18). Some studies found relationships between levels of urinary mercury (from both occupational and dietary exposure) and serum prolactin (18). However, the behavior of these relationships was not always the same, perhaps because of a different influence for each mercury compound (18). Therefore, it is essential to identify the factors controlling prolactin secretion.

Thus, in this work, one of the major cellular mechanisms of MeHg toxicity (oxidative stress produced by free radical generation) was studied to analyze its influence on prolactin release in cells of pituitary origin. MeHg is able to increase the generation of free radicals (highly reactive molecules with only a single electron in the highest electronic energy level) in many tissues (16). Actually, MeHg leads to activation of NOS (a key enzyme that synthesizes nitric oxide, a reactive oxygen species), leading to an increase in production of free radicals (2). Some studies have already demonstrated the protective effect exerted by antioxidants, such as vitamin C or melatonin (19), against mercury toxicity. Interestingly, inhibition of NOS by L-NARG completely prevented the decrease in prolactin release provoked by MeHg, including when a higher concentration and a longer time of exposure were used (Figure 3, top panel). This strong relationship supports the idea that the MeHg effect on prolactin production may be mediated via free radical production and, especially, via activation of the nitrergic system. However, additional studies are being conducted to clarify whether MeHg actually activates NOS enzymes in GH3B6 cells, to eliminate the possibility that an intrinsic NOS activity, not affected by MeHg, may participate in the MeHg-induced inhibition of PRL release.

In this study, we observed that treatment of GH3B6 cells with L-NARG did not alter the basal prolactin release; similar results have been described by Chiodera et al. (20). The authors showed that treatment of humans with L-NAME, an inhibitor of NOS, does not affect basal release of prolactin, but may cause an increase in prolactin release induced by vasoactive intestinal peptide (VIP). Thus, we believe that basal NOS activity does not interfere significantly with basal prolactin release, but the activation of this enzyme in GH3B6 cells induced by MeHg is an important inhibitory modulator of prolactin release. These effects may prove to be useful tools for elucidating the mechanisms by which prolactin release can be controlled.

The maintenance of prolactin secretion levels after exposure to MeHg, due to inhibition of NOS, occurred without an acute neurotoxic effect since no significant difference was detected in cellular viability for groups incubated with 1 or 10 μM MeHg (Figures 2 and 3, bottom panel). Thus, the decrease in prolactin production appears to be an early effect of MeHg in cells of pituitary origin. Taking into account the versatility of MeHg, other mechanisms of mercury toxicity, such as microtubule disruption, may also be participating simultaneously, because disassembly of tubulin microtubules could affect vesicle transport of prolactin. However, preliminary results of studies carried out with the same low doses and short exposure times used in this work indicated that microtubule modification would be minimal in these conditions (data not shown), suggesting that the nitrergic system is the major system responsible for the decrease of prolactin release because of MeHg exposure.

## Supplementary material


