# Supplementary material for: Methylmercury inhibits prolactin release in a cell line of pituitary origin
Source: Braz J Med Biol Res. 2015 Jun 23;48(8):691–6. doi: 10.1590/1414-431X20154165 (PMC4541687; doi:10.1590/1414-431X20154165)

Figure S1. Cellular viability of the rat pituitary cell line GH3B6 exposed to different methylmercury concentrations for 2 h (*closed triangles*) or 6 h (*closed squares*). Data are reported as means±SE (n=6). ***P<0.001 *vs* all groups (ANOVA with Tukey's test).

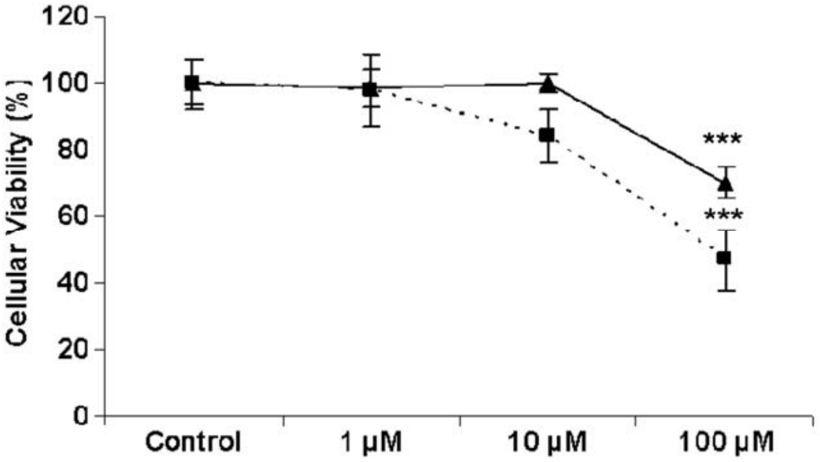

Supplement: Supplementary file 1 [file 1414-431X-bjmbr-48-08-00691-suppl001.pdf]
